# Prenatal sonographic diagnosis of pseudomonoamniotic twins: Two-case series

**DOI:** 10.1097/MD.0000000000046533

**Published:** 2025-12-12

**Authors:** Fengbei Kong, Guoru Wu, Desheng Sun, Ting Qin, Yue Wang, Yixin Zhu, Ran Chen

**Affiliations:** a Department of Ultrasonography, Peking University Shenzhen Hospital, Shenzhen, Guangdong Province, China.

**Keywords:** amniotic band syndrome, dividing-membrane rupture, prenatal diagnosis, pseudomonoamniotic twins, ultrasonography, umbilical cord entanglement

## Abstract

**Rationale:**

Pseudomonoamniotic twins (PMA), a rare and high-risk complication of diamniotic twins, result from the rupture of the dividing membrane. This condition is associated with fetal malformations and demise. Current prenatal ultrasound detection rates remain suboptimal, with most cases diagnosed only after umbilical cord entanglement or intrauterine fetal death.

Here, we present 2 cases of PMA diagnosed prenatally via ultrasound and focus on discussing their sonographic features.

**Patient concerns:**

Case 1: No first-trimester ultrasound was performed in this case. The patient visited our hospital for the first ultrasound examination at 12weeks’ gestation.

Case 2: Follow-up ultrasound was performed 1 day after amniocentesis at 26 weeks’ gestation.

**Diagnoses:**

Case 1: PMA with amniotic band syndrome.

Case 2: PMA with cord entanglement and fetal distress.

**Interventions:**

Case 1: The twins did not receive any specific treatment.

Case 2: Emergency cesarean delivery was performed.

**Outcomes:**

Case 1: Induction of labor at 13 weeks’ gestation.

Case 2: Preterm twins admitted to neonatal intensive care unit, discharged after 50 days with favorable outcomes.

**Lessons:**

Targeted evaluation of the dividing membrane at the umbilical cord insertion sites may improve the detection of spontaneous PMA. Sonographic manifestations of dividing-membrane rupture include the multilayer folding sign, absence of the dividing membrane, 1 fetus entering the amniotic sac of the co-twin, and umbilical cord entanglement. The multilayer folding sign may be an early indicator of the rupture dividing membrane. Complications resulting from dividing-membrane rupture warrant heightened vigilance, especially amniotic band syndrome and cord entanglement, both of which are associated with adverse perinatal outcomes.

## 1. Introduction

Since the 1980s, the global twinning rate has increased by one-third, rising from 9.1 to 12.0 per 1000 deliveries, primarily attributable to the widespread adoption of assisted reproductive technologies, family history, and increased maternal age, resulting in approximately 1.6 million twin births annually.^[1]^ Monoamniotic twin pregnancy occurs in approximately 1% to 2% of monozygotic gestations and carries the highest risk of perinatal morbidity and mortality, with a perinatal mortality rate of 10% to 30%. This risk may be secondary to prematurity, growth restriction, congenital anomalies, vascular anastomoses, and, most commonly, umbilical cord entanglement, which is reported to occur in as many as 70 % of monoamniotic twins.^[2,3]^ Suzuki retrospectively evaluated a series of 18 monoamniotic and 7 pseudomonoamniotic twins (PMA). No significant differences were observed in the incidence of neonatal death or umbilical cord entanglement between the 2 groups.^[4]^

PMA is a unique form of twin gestation, pathologically characterized by the rupture of the dividing membrane, leading to the merging of 2 originally separate amniotic cavities into a single compartment. Most cases occur in monochorionic diamniotic twins. Jeanty reported the first spontaneous PMA in dichorionic diamniotic twins in 2010.^[5]^ After searching PubMed, Ovid, and Chinese Core Journals, we identified, since 1991,48 reported cases of spontaneous PMA and more than 100 cases of iatrogenic PMA. Current studies on PMA twins cover etiology, diagnosis, and management strategies; however, the exact cause of spontaneous PMA remains unknown.^[6–8]^ However, due to the rupture of the dividing membrane, approximately 44% of cases result in adverse pregnancy outcomes, primarily fetal malformations caused by amniotic band syndrome (ABS) and fetal demise resulting from cord entanglement.^[9–12]^ Antenatal sonographic diagnosis is an important tool for detecting PMA, enabling timely obstetric management and reducing perinatal mortality risks.^[13]^ Nevertheless, the thin dividing membrane and other factors make early recognition of membrane rupture a significant diagnostic challenge.

Here, we report 2 PMA cases diagnosed at different gestational ages and associated with different adverse outcomes. We focused on sonographic features of the dividing-membrane rupture.

## 2. Case reports

**Figure 1.**
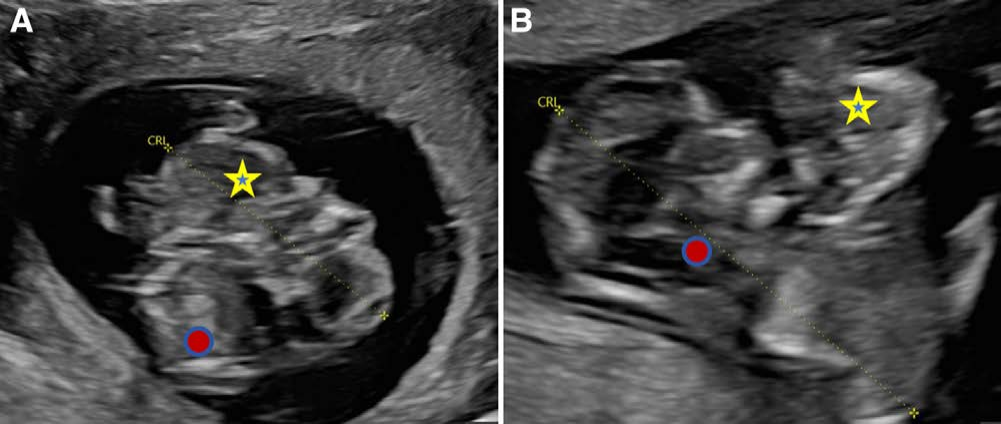
Transabdominal scan demonstrates that fetal A’s abdomen is continuously adjacent to fetal B’s thorax and reveals abnormal spinal morphology in fetus A. (A) Long-axis view of fetus A. (B) Long-axis view of fetus B. Asterisk denotes fetus A; circle denotes fetus B.

**Figure 2.**
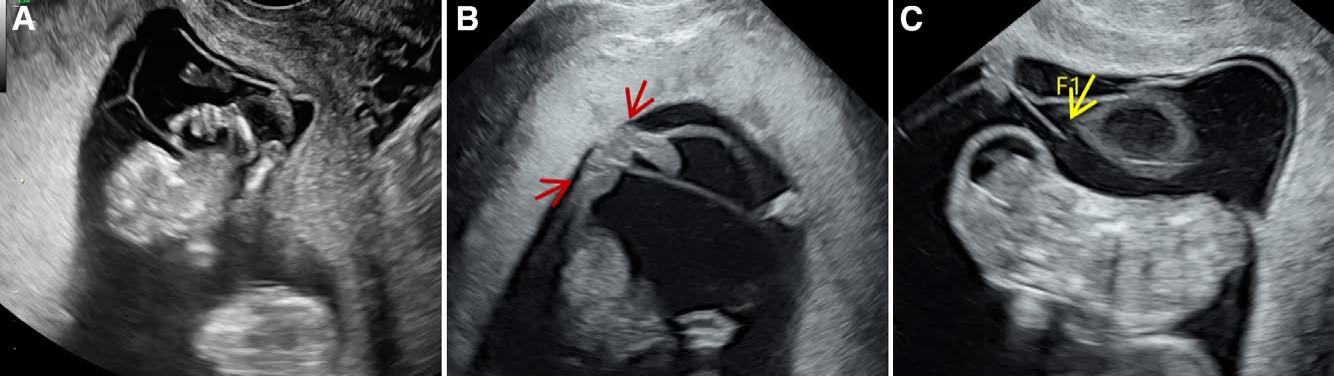
Transvaginal scan. (A) The body of fetus A enters the amniotic sac of fetus B. (B) Two umbilical cord insertion points are in close proximity. (C) Membrane rupture is observed 2 cm from the insertion point. Red arrowheads indicate the umbilical insertion points; the yellow arrowhead indicates the residual free edge of the ruptured dividing membrane.

Case 1: A 31-year-old primigravida who conceived spontaneously presented for a prenatal ultrasound at 12 weeks’ gestation. Earlier screenings were missed because the pregnancy was initially unrecognized. Transabdominal sonography was performed initially. Ultrasound images revealed a monochorionic twin pregnancy with an indistinct dividing membrane. Additionally, multiple anomalies, including local cranial defects, abnormal spinal curvature, and disrupted skin continuity over the spine, were noted. Throughout the examination, the chest of fetus B remained continuously adhered to the abdomen of fetus A without any separation, even under gentle transducer pressure (Fig. 1). The dividing membrane was not completely visualized. Subsequently, transvaginal sonography revealed partial herniation of Fetus A into the amniotic sac of Fetus B, with the fetus remaining fixed in this position (Fig. 2A). Two umbilical cord insertion points were in close proximity (Fig. 2B), and membrane rupture was identified 2 cm from these points (Fig. 2C). Residual membrane fragments were observed on the skull surface, abdomen, and limbs of Fetus A. These findings indicate that dividing-membrane rupture leads to ABS. After comprehensive counseling, the patient opted for pregnancy termination.

**Figure 3.**
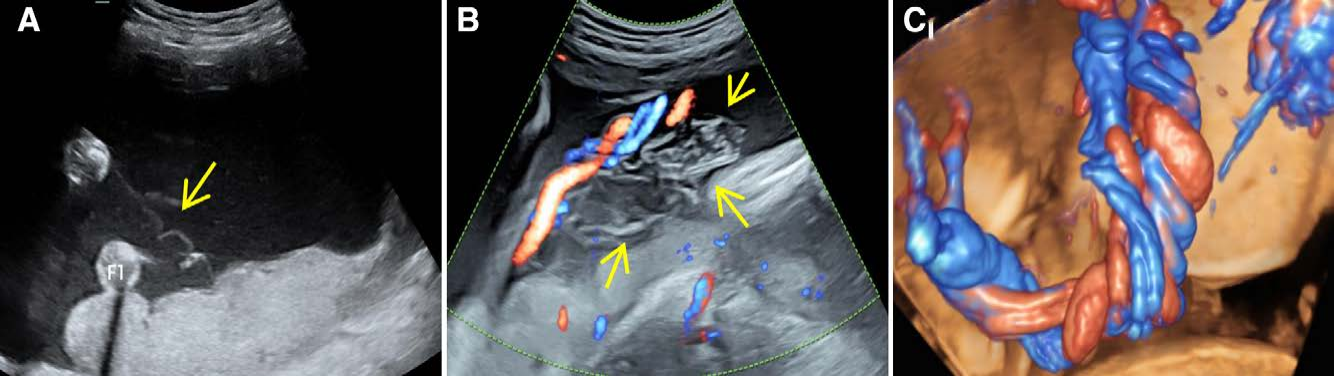
(A) At 26 wk, subtle multilayer folding is evident. (B) At 28 wk, the multilayer folding sign becomes typical.(C) At 31 wk, 3-dimensional color Doppler demonstrates cord entanglement.The yellow arrow points to the multilayer folding sign, representing the ruptured and collapsed dividing membrane.

Case 2: A 36-year-old woman (G2P1) was diagnosed with monochorionic diamniotic twins at 8 weeks of gestation. Selective intrauterine growth restriction developed in one of the fetuses at 24 weeks. The maximum amniotic fluid depths were 26 mm (sac A) and 65 mm (sac B), respectively. Amniocentesis was performed at an outside hospital at 26 weeks’ gestation. One day post-procedure, the woman presented to our hospital for a follow-up ultrasound. The images show poorly visualized segments of the dividing membrane. We initially suspected that the membrane was closely apposed to the fetal body due to oligohydramnios. After maternal position changes and gentle fetal manipulation, amniotic fluid spaces appeared between the fetus and the uterine wall. Real-time ultrasound showed both fetuses moving freely, effectively excluding the “stuck-twin” appearance characteristic of twin-to-twin transfusion syndrome. Concurrently, we observed that membrane tension was reduced and subtle folding (Fig. 3A). Considering the recent invasive procedure, we discussed the risk of dividing-membrane rupture with the patient, although no definitive tear was identified. At the 28-week follow-up scan, we observed a torn dividing membrane with disorganized redundant folds accumulated along the rupture site (Fig. 3B), confirming definitive membrane rupture and establishing a diagnosis of PMA. The woman underwent weekly checkups. At 31 weeks, cord entanglement was detected (Fig. 3C), whereas the Doppler waveforms of the umbilical artery remained normal. She requested inpatient observation. At 33 weeks, 1 fetus exhibited an elevated umbilical artery pulsatility index and severe variable decelerations on the nonstress test (NST). Therefore, an emergency cesarean was performed. Intraoperative examination confirmed membrane rupture and cord entanglement. Twin A weighed 1530 g with Apgar scores of 8 and 9 at 1 and 5 minutes, respectively. Twin B weighed 1820 g with Apgar scores of 5 and 8at 1 and 5 minutes, respectively. Both infants were admitted to the the neonatal intensive care unit(NICU) and discharged after 50 days with favorable outcomes.

## 3. Discussion

### 3.1. Pathogenesis of PMA

The etiology of membrane rupture in PMA includes both iatrogenic and spontaneous causes. The mechanisms of spontaneous rupture remain incompletely understood; however, potential contributors encompass congenital membrane defects, chorioamnionitis, fetal hyperactivity.^[6,14–16]^ Iatrogenic rupture is most commonly associated with invasive procedures, notably amniocentesis, septostomy, laser surgery.^[17,18]^ In Case 1, despite lacking early evidence of diamniotic twins, we considered this case to represent PMA based on the residual dividing membrane. We speculated that the cause of membrane rupture may be congenital perforation or spontaneous rupture occurring before 12 weeks’ gestation. In addition, Lee et al proposed that a short distance between umbilical cord insertions (typically < 5 cm apart) may lead to spontaneous PMA.^[19]^ This may be because vascular anastomosis causes blood flow diversion and insufficient perfusion of the dividing membrane, thereby increasing its fragility. This hypothesis has been corroborated by multiple case reports.^[14,20–22]^ Our case, which featured closely approximated cord insertion sites, provides further evidence supporting this hypothesis.

### 3.2. Diagnosis of PMA

Prenatal diagnosis of **PMA** primarily relies on ultrasonography. Antenatally diagnosed cases are associated with lower rates of adverse pregnancy outcomes than those diagnosed postnatally. Nevertheless, the early sonographic detection of dividing-membrane rupture remains challenging. Fleming and Miller found that only 60% of cases were diagnosed prenatally, and the majority of cases were identified only after cord entanglement or even fetal demise.^[9]^ Ultrasound diagnosis is based on the early confirmation of diamniotic twins, which later turned out to be monoamniotic twins in subsequent examinations. The sonographic manifestations of dividing-membrane rupture mainly include the absence of the dividing membrane, 1 fetus entering the amniotic sac of the co-twin, and umbilical cord entanglement.^[22,23]^ Chadha et al first reported the multilayer folding sign as an ultrasonographic indicator of dividing-membrane rupture.^[14]^ However, this sign has not yet received adequate attention. In our cases, the dividing membrane was not completely absent, which made diagnosis difficult. However, we correctly diagnosed PMA using the other 3 ultrasound signs. In particular, the multilayer folding sign, which helped us suspect membrane rupture early, was a key early diagnostic clue. This sign probably results from decreased membrane tension after rupture, leading to incomplete retraction. However, it should be noted that false positives or false negatives may occur when diagnosing PMA. A dividing membrane can be identified in only 90 % of diamniotic pregnancies, which gives a false impression of a monoamniotic twin pregnancy.^[24]^ In such cases, high-frequency ultrasonography is recommended to reassess the integrity of the thin membrane. When the incision is small or the fetal body obscures the dividing membrane, PMA can easily be missed. In Case 1, the residual dividing membrane adhered to the fetal surface with adequate tension. Therefore, no classic multilayer folding sign was developed. In certain sectional views, the membrane appeared intact, and multiple malformations were observed. Therefore, this condition can be easily misdiagnosed as simple ABS. During umbilical cord insertion site scanning, we observed that the twin insertion points were in close proximity and a large area of the dividing membrane near the insertion point was absent. A part of the fetal body entered the other amniotic sac through the defect. These signs helped us to quickly diagnose the rupture of the dividing membrane. As previously mentioned, some studies have reported cases of spontaneous rupture of the dividing membrane with closely spaced umbilical cord insertion points, and histopathological examinations have revealed membrane rupture adjacent to these sites. Combined with these findings, a systematic ultrasound assessment of the dividing-membrane integrity near the cord insertion sites may facilitate the early detection of spontaneous rupture. Case 2 illustrates this progressive process in detail. On the initial scan, we considered that the dividing membrane might be adherent to the fetal body secondary to oligohydramnios, leading to an incomplete view of the so-called “stuck-twin” phenomenon, which is seen in severe twin-to-twin transfusion syndrome.^[25]^ To avoid misdiagnosis in this scenario, it is very important to visualize the whole membrane as much as possible by changing the position of the mother, gentle fetal manipulation, and multiplanar scanning.

### 3.3. Complications of dividing-membrane rupture

The complications of dividing-membrane rupture mainly include preterm birth, ABS, and cord entanglement. Fayez et al reviewed 16 PMA cases and reported umbilical cord entanglement in 56 % of fetuses and a 44 % fetal mortality rate.^[26]^ Suzuki pointed out that there was no significant difference in perinatal fetal loss between monoamniotic twins and PMA.^[4]^ Therefore, the same serious management may be required for PMA as for monoamniotic twins. Dividing-membrane rupture can also lead to ABS. Most cases occur after invasive procedures in diamniotic twins, with a reported incidence of 1.8 to 3.3 %.^[18,27]^ Hvelplund et al reported the first case of spontaneous PMA with fatal ABS diagnosed postpartum, in which 1 fetus died.^[15]^ Based on our and previously reported cases, dividing-membrane rupture is strongly associated with adverse perinatal outcomes.

## 4. Conclusions

PMA carries a significant risk of disability and mortality. Therefore, we recommend a thorough assessment of the dividing-membrane integrity, particularly around the cord insertion sites, during routine ultrasound examination of diamniotic twin gestation. Familiarity with its sonographic features and common diagnostic pitfalls will facilitate the accurate prenatal recognition of PMA. Whenever rupture is suspected, active screening for complications of ABS and cord entanglement is necessary.

## Acknowledgments

The authors are deeply thankful to Director Tirong Zhang for his invaluable assistance in the prenatal diagnosis of these complex cases, providing high-resolution ultrasound images.

## Author contributions

**Conceptualization:** Fengbei Kong, Guoru Wu.

**Data curation:** Ting Qin, Ran Chen.

**Funding acquisition:** Desheng Sun.

**Investigation:** Guoru Wu.

**Supervision:** Yue Wang.

**Writing – original draft:** Fengbei Kong.

**Writing – review & editing:** Fengbei Kong, Yixin Zhu.
